# First Insight into the Prevalence of *Coxiella burnetii* Infection among Veterinary Medicine Students in Bulgaria

**DOI:** 10.3390/idr16050061

**Published:** 2024-08-26

**Authors:** Petia Genova-Kalou, Yordan Hodzhev, Ilia Tsachev, Roman Pepovich, Stefan Panaiotov, Veselin Dobrinov, Stefka Krumova, Betina Boneva-Marutsova, Borislava Chakarova, Keytlin Todorova, Konstantin Simeonov, Magdalena Baymakova, Pierre-Edouard Fournier

**Affiliations:** 1 Department of Virology, National Center of Infectious and Parasitic Diseases, 1233 Sofia, Bulgaria; 2 Department of Microbiology, National Center of Infectious and Parasitic Diseases, 1504 Sofia, Bulgaria; 3 Department of Microbiology, Infectious and Parasitic Diseases, Faculty of Veterinary Medicine, Trakia University, 6000 Stara Zagora, Bulgaria; 4 Department of Infectious Pathology, Hygiene, Technology and Control of Foods from Animal Origin, Faculty of Veterinary Medicine, University of Forestry, 1797 Sofia, Bulgaria; 5 Department of Hygiene, Epidemiology, Microbiology, Parasitology and Infectious Diseases, Faculty of Medicine, Trakia University, 6000 Stara Zagora, Bulgaria; 6 National Diagnostic and Research Veterinary Medical Institute “Prof. Dr. G. Pavlov”, Bulgarian Food Safety Agency, 1606 Sofia, Bulgaria; 7 Department of Infectious Diseases, Military Medical Academy, 1606 Sofia, Bulgaria; 8 French Reference Center for Rickettsioses, Q Fever and Bartonelloses, IHU-Méditerranée Infection, 13005 Marseille, France

**Keywords:** *Coxiella burnetii*, Q fever, veterinary medicine students, Bulgaria

## Abstract

The aim of this study was to assess the prevalence of *Coxiella burnetii* infection among veterinary medicine students from two Bulgarian Universities, located in Sofia and Stara Zagora. Blood samples were collected from a total of 185 veterinary students for the detection of *C. burnetii* phase II antibodies and presence of DNA using an enzyme-linked immunosorbent assay (ELISA) and end-point PCR test. Out of all samples, 29.7% were positive for at least one *C. burnetii* phase II antibody marker or by the result of the PCR test. Veterinary students from Stara Zagora showed a significantly high seropositivity for Q fever (33.6%), as compared to the students in Sofia (23%; *p* < 0.05). Evidence of recent exposure with detection of anti-*C. burnetii* phase II IgM (+) antibodies was observed in 14.6% of the students under study. Seroprevalence among students in Stara Zagora was higher (15.3%). Anti-*C. burnetii* phase II IgG antibodies were detected in 21.6% of examined samples. Our study revealed a higher seropositivity among the male students (32.8%) as compared to females (16.0%; *p* < 0.05). The end-point PCR assay detected 5.9% blood samples as positive. The relative risk (RR) of Q fever exposure for male students was 40.7%, whereas it was 24.6% in females (*p* < 0.05). The findings from this study indicate that the *C. burnetii* infection is widely distributed amongst veterinary students in Bulgaria. This study emphasizes the need for improved safety protocols and infection control measures in veterinary training programs.

## 1. Introduction

Q fever is an endemic zoonosis of global distribution, caused by the intracellular bacterium *Coxiella burnetii*, which is of particular significance to human and animal health [1,2,3]. Over the last few years, the highest numbers of confirmed cases of Q fever in Europe have been reported in Spain, France, Germany, Romania, Hungary and Bulgaria [4,5,6,7,8,9]. The primary sources of human infection are domestic ruminants, such as goats, cattle and sheep, and pets, in which the infection is usually asymptomatic, but may also be associated with infertility, abortions and premature birth [10,11,12,13]. The clinical manifestations of Q fever in humans varies greatly, from a nonspecific, self-limiting disease to an acute form (fever of unknown origin, atypical pneumonia, granulomatous hepatitis) [14,15,16] or may have chronic presentation (endocarditis, vascular infection, osteomyelitis, etc.) [17,18]. The laboratory diagnosis of Q fever is based on (i) isolation and cultivation, which requires BSL3 facilities for tissue cultures [19]; (ii) serological tests, such as indirect immunofluorescence (IFA), which is considered to be the gold standard and enzyme-linked immunosorbent assay (ELISA) [20,21]; (iii) antigen detection assays, such as immunohistochemical staining (IHC) [22]; and (iv) molecular detection of *C. burnetii* DNA by different PCR techniques for early diagnosis of the acute phase [23,24]. In acute Q fever, the anti-*C. burnetii* antibody response is detectable in the 2–3 weeks following infection. The level of anti-*C. burnetii* phase II IgM antibodies increases rapidly after infection, and they are detectable for several months as a marker of a current infection. The presence of specific phase II IgG antibodies provides evidence of a recent *C. burnetii* infection or a past exposure to the pathogen, which can remain detectable for years [25,26].

Due to the fact that *C. burnetii* is environmentally stable and can be transmitted from animal hosts to humans mainly via the inhalation of contaminated aerosols or dust particles [27,28], the most vulnerable people are professionals who have daily contact with farm animals and pets and are therefore at a higher occupational risk of exposure [29,30,31]. Veterinary medicine students remain at high risk during their six-year veterinary curriculum, since their training includes animal handling and the treatment of a wide variety of species [32]. According to the scientific literature, few serological studies have focused on *C. burnetii* seroprevalence among veterinary medicine students [33,34,35]. So far, only a few studies have assessed the zoonotic risks for veterinary medicine students [36,37,38].

Q fever is considered endemic for Bulgaria; however, due to its nonspecific and pleomorphic clinical presentation, Q fever often remains neglected and underdiagnosed. Thus, little is known of Q fever seroprevalence among various professional groups occupationally exposed to *C. burnetii* infection, including veterinary medicine students, because of their close contact with infected livestock and pets during their curricular trainings.

Thus, the aim of the present study was to assess the prevalence of *C. burnetii* infection among veterinary medicine students at two Veterinary Medicine Faculties (in Sofia and Stara Zagora), located in the Western and Central parts of the country. To the best of our knowledge, this is the first evidence of *C. burnetii* infection in veterinary medicine students in Bulgaria.

## 2. Materials and Methods

### 2.1. Target Population and Sample Collection

**Figure 1:**
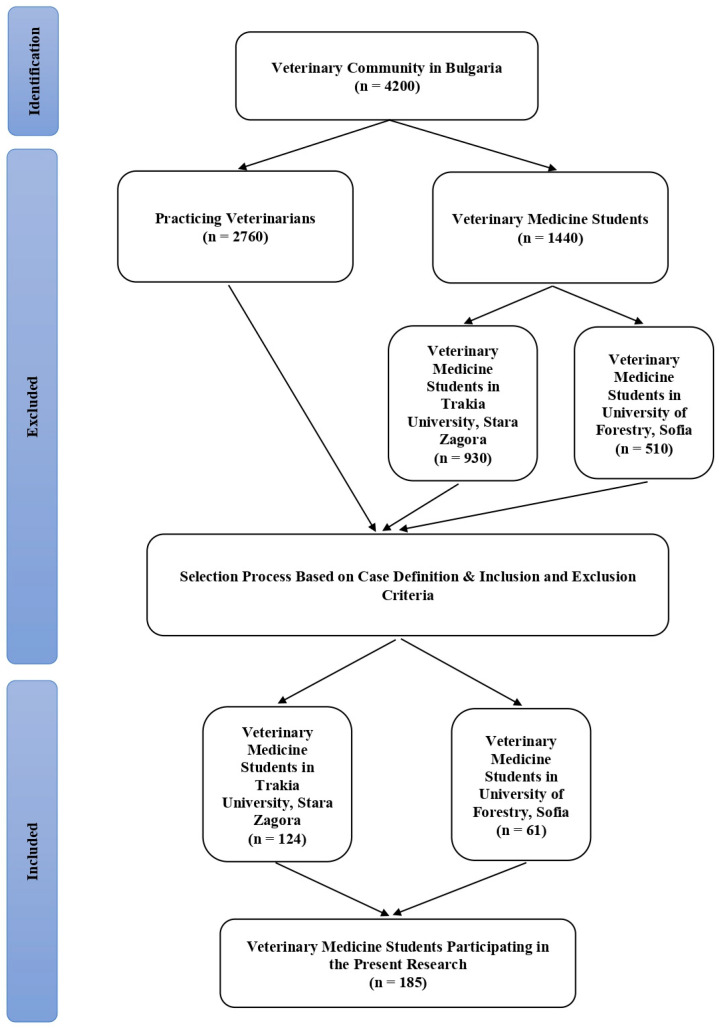
Flow chart showing the selection process of research participants.

In this study, blood samples were taken from a total of 185 veterinary medicine students in their 5th year of study during the 2022–2023 academic year at the two Veterinary Faculties of the University of Forestry in Sofia and Trakia University in Stara Zagora, Bulgaria. The participants in the present research were selected on the basis of case definition, as well as inclusion and exclusion criteria (Figure 1).

The students, included in this study had to meet the following criteria: (a) age > 18 years; (b) students in the 5th year of study and majoring in “Veterinary Medicine”; (c) participants at the end of their cursus who were in close contact with farm animals and pets during practical training; (d) participants who gave a submission of a written informed consent for participation in the research survey; (e) female participants who did not report a possible pregnancy at the moment of the survey. The exclusion criteria for the present research included the following: (a) individuals younger than 18 years; (b) persons from other university specialties, such as mathematics, engineering, finance and economics, law, and human sciences; (c) individuals with contraindications for venipuncture; (d) persons who refused to give written informed consent; (e) participants with a Q fever vaccination history; (f) persons with a severe clinical form of an acute or chronic disease; (g) students who were pregnant.

The samples from the students were obtained during routine diagnostic tests following a standard procedure. After obtaining a written consent and completing a questionnaire, students were invited to donate a blood sample of 10 mL by venipuncture for testing. The blood samples were delivered to the National Reference Laboratory (NRL) “Rickettsia and cell cultures”, Department of Virology, National Centre of Infectious and Parasitic Diseases, Sofia (Bulgaria). The sera were separated by centrifugation at 1500 rpm for 10 min, transferred into sterile vacuum tubes and stored at −20 °C until processing. Whole blood samples (200 µL), collected in EDTA tubes, were used for DNA extraction.

### 2.2. Case Definition

The presence of anti-*C. burnetii* phase II IgG antibodies was assumed to indicate an acute *C. burnetii* infection or a history of a past one, i.e., a previous encounter with this pathogen. *C. burnetii* infection was defined as acute when one of the following criteria was present: (a) a 4-fold increase in anti-*C. burnetii* phase II IgG antibody titers specific to *C. burnetii* between paired serum samples, as evidenced by an ELISA; (b) a positive ELISA result for anti-C. burnetii phase II IgM antibodies; (c) the presence of *C. burnetii* DNA detected in EDTA blood specimens via the amplification of a specific target by PCR assay, <21 days following the onset of the disease; (d) a negative blood PCR result but a positive ELISA result for IgM and IgG antibodies against phase II antigens of *C. burnetii*. Paired sera samples taken 3 to 6 weeks and demonstrating a 4-fold or greater rise in *C. burnetii* phase II IgG antibody titers between the acute and convalescent phase confirmed the diagnosis of acute Q fever.

### 2.3. Serological Tests

The serum samples were examined for the presence of anti-*C. burnetii* phase II IgM and IgG antibodies using a commercial ELISA kit (EUROIMMUN, Lübeck, Germany) according to the manufacture’s recommendations. The absorbance values were determined at 450 nm (reference wavelength of 620–690 nm) using an Epoch ELISA reader (Agilent BioTek Epoch Microplate Spectrophotometer, Agilent Technologies, Santa Clara, CA, USA), on the Gen5 version 2.06 analysis software (BioTek Instruments, Inc., Winooski, VT, USA). The optical density (OD), cut-off values and controls were recorded, respectively. The results were evaluated semi-quantitatively by calculating the ratio (R) of the extinction value of the patient’s sample over the extinction value of the calibrator. The results were categorized as follows: R ≥ 1.1 was accepted as positive indicating a possible acute infection or evidence of past one; R < 0.8 was considered negative; and R ≥ 0.8 to <1.1 indicated a borderline result [39].

### 2.4. PCR Assay

Whole blood samples were tested for the presence/absence of *C. burnetii* DNA using end-point PCR and specific primers. Genomic DNA was extracted using the QIAamp DNA Mini Kit (cat. No 51304, Qiagen, Hilden, Germany) according to the manufacturer’s instruction. DNA was eluted with forty microliters of elution buffer and stored at 4 °C until use. The primers were designed to amplify a 687 bp fragment of the transposon-like repetitive region of the bacterial genome (*IS1111*) of *C. burnetii*, using the Trans-1 and Trans-2 primers with the following sequence: Trans-1 (5′-TAT GTA TCC ACC GTA GCCAGT C-3′) and Tans-2 (5′-CCC AAC AACACC TCC TTATTC-3′) (Metabion, Planegg, Germany) [40]. The Trans-PCR was carried out as described [41]. The resulting amplicons underwent electrophoresis on 1.5% agarose gel and visualization under UV light [42].

### 2.5. Statistical Analysis

Data analysis was performed using the SPSS Statistics 26.0 (IBM Corp., Armonk, NY, USA) and Excel 2016 (Microsoft, Redmond, WA, USA) software platforms. The data were entered and arranged in MS Excel. The risk exposure to *C. burnetii* of the different groups of veterinary students was calculated, as categorized by sex and location. The statistical analysis compared the relative risks (RRs) as the proportion of the exposed over unexposed students. A Chi square test was utilized for this comparison, and a *p*-value < 0.05 was considered statistically significant. The final results obtained from the statistical analysis were confirmed by all scientists involved in this study.

### 2.6. Ethical Considerations

All veterinary medicine students signed a written informed consent for participation in the research. Participants gave written informed consent for the invasive procedures conducted on them (venipuncture). Students had individual access to his/her laboratory result. The research was conducted in accordance with the ethical principles of the Declaration of Helsinki (adopted in June 1964, last revision October 2013). The present survey was approved by the Local Ethics Committee of the National Centre of Infectious and Parasitic Diseases, Sofia, Bulgaria (NCIPD-02/7 February 2023), who confirmed that the research was in full accordance with all ethical principles and practices. The authors have not used artificial intelligence (AI) to create this manuscript.

## 3. Results

### 3.1. Characterization of the Study Population

A two-step approach was used to distinguish between a present acute *C. burnetii* infection from a past one. First of all, all serum samples were screened by a commercial ELISA kit for the presence of anti-*C. burnetii* phase II IgM and IgG antibodies. Then, the positive samples were analyzed by an end-point PCR assay, targeting *IS1111* transposable element, which is routinely used as a marker in the epidemiological investigation of *C. burnetii* infections.

### 3.2. Serology and PCR Analysis

Table 1 shows the distribution of participants according to their serological result, PCR results, university and gender. The categories include combinations of IgM, IgG and PCR results, indicating different stages and types of exposure to or infection with *C. burnetii*. Among the 185 serum samples studied, 55 (29.7%) were positive for at least one marker of the anti-*C. burnetii* phase II antibody or for the presence of *C. burnetii* DNA (Chi square (1) = 27.88; *p* < 0.001). Veterinary medicine students from Stara Zagora showed significantly more seropositivity against *C. burnetii* as compared to those from the capital city of Sofia (42 out of 125 (33.6%) vs. 14 out of 61 (23%), respectively, Chi square (1) = 2.22; *p* < 0.05) Stratified by gender, 24 of 59 (40.7%) male samples were positive, versus 31 of 126 (24.6%) female samples (Chi square (1) = 4.00, *p* < 0.05).

Among all veterinary students tested who provided a serum sample, 28 (14.6%) had evidence of recent exposure (presence of IgM to phase II *C. burnetii*). Of those, 4 samples (14.8%) were obtained from males and 23 (85.2%) were obtained from females (Chi square (1) = 3.98, *p* < 0.05). Overall, 8 of 61 serum samples (13.1%) from veterinary students in Sofia were positive for phase II IgM antibodies. The seroprevalence among students in Stara Zagora was 15.3% (19/126) (Chi square (1) = 0.14, *p* = 0.71).

Anti-*C. burnetii* phase II IgG antibodies were detected in 40 of the 185 (21.6%) serum samples examined. The seroprevalence for phase II IgG antibodies observed in our study population was 14.8% and 25% for University of Forestry and Trakia University, respectively; however, the result obtained was not significant (Chi square (1) = 2.44, *p* > 0.1). It should be noted that the frequency of positive serum titers for Q fever (phase II) was greater in males than in females (Chi square (1) = 6.84, *p* < 0.01). Moreover, the incidence of seropositivity for IgG was almost twice in males (39%) than in females (13.5%) (Chi square (1) = 6.84, *p* < 0.01). Out of the 10 patients with suspected acute Q fever infection (presence of anti-*C. burnetii* phase II IgM/IgG (+) antibodies), the proportion in females was higher than that in males.

### 3.3. Molecular Analysis

EDTA blood samples were further screened in this study by end-point PCR assay for the detection of *C. burnetii* DNA. The end-point PCR detected 11 out of 185 (5.9%) blood samples as positive at 687 bp by Trans-1 and Trans-2 primers. In general, 7.1% of the females and 3.4% of the males tested positive.

### 3.4. Relative Risk Assessment of Q Fever Exposure

The relative risk (RR) of Q fever exposure among veterinary students was assessed by comparing the prevalence of anti-*C. burnetii* antibodies between the different groups, specifically focusing on sex and university affiliation (Table 2). For clarity, all cases that showed at least one positive marker (IgG, IgM or PCR) were pooled together and designated as “exposed” and were compared to the number of negative cases (“unexposed”). The relative risk of Q fever exposure for the students in Stara Zagora and Sofia was 33.1% and 23%, respectively. The relative risk (RR) of Q fever exposure for male students was 40.7% and 24.6%, respectively. A location-by-sex RR analysis showed that the relative risk of Q fever exposure for male students in Stara Zagora was 50%, followed by female students in Stara Zagora (25.6%). Male and female veterinary students in Sofia had an RR of 23.8% and 22.5%, respectively.

## 4. Discussion

Q fever is among the most common zoonotic diseases, and even in countries with limited information, the available data show a high prevalence of infection in people who are professionally involved in animal husbandry [43]. The results of our study are in agreement with similar data reported by other authors in Europe. In 2000, Valencia et al. reported a 11.02% *C. burnetii* seroprevalence in veterinary students from Zaragoza, Spain [38]. In Turkey, seroprevalences of anti-*C. burnetii* antibodies of 7.0% and 8.0% were detected among 83 veterinarians from two distinct geographic regions [44]. German scientists detected 38.0% positive sera for IgG to phase II *C. burnetii* among 424 veterinarians [45]. In 2012, de Rooij et al. reported anti-*C. burnetii* IgG in Dutch veterinary medicine students [36]. One year later, another Dutch scientific team found antibodies against *C. burnetii* among 65.1% of 189 livestock veterinarians [46]. Similar reports have been publish by other authors as well [47,48,49]. In short, our results and data from other authors throughout Europe show a moderate to high level of *C. burnetii* seropositivity among risk groups (persons in contact with animals; predominantly veterinarians).

To assess the importance of *C. burnetii* infection among risk groups (veterinarians, hunters, etc.), it is recommended to make a comparison with the seropositivity in the general population. In Tuscany, Tiscione et al. reported lower levels of *C. burnetii* antibodies among an urban group with no potential animal contact (6.1%) compared to the higher levels, detected in a rural group with higher potential exposure to animals (49.1%) [50]. A few years later, Pascual Velasco et al. identified 3.0% of seropositive individuals in the healthy population from Lanzarote, Canary Islands [51]. In Northern Ireland, McCaughey et al. detected a 12.8% seropositivity among 2394 randomly selected subjects [52]. By testing plasma samples obtained over time in Southern Netherlands, Brandwagt et al. [53] reported significant dynamics in Q fever seroprevalence levels, ranging from 62.5% in 1983 to 14.4% in 1987, and decreasing to 1% in 2008, with a slight increase in 2.3% in 2010. Similar data were also reported by other authors [54,55,56,57]. These data clearly demonstrated a lower seropositivity for *C. burnetii* in the general population compared to the risk groups. This confirms the role of this bacterium as an occupational disease pathogen and highlights the need for more detailed epidemiological investigations in exposed population.

In our survey, high seroprevalence rates against *C. burnetii* (30.8%) were found among veterinary medicine students. The potential reasons for this situation may be multiple and of different natures. Q fever is endemic in Bulgaria, and high environmental contamination can be expected due to the wide prevalence of *C. burnetii* infection found amongst ruminant herds/flocks in the country (Simeonov K, unpublished data). In addition, *C. burnetii* infection is easily acquired by inhalation of contaminated aerosols, which facilitates the transmission to people and the spread of the bacterium [58,59]. *Coxiella burnetii* can withstand long periods in harsh environments [60] and the infected dust is contagious and can blow far from the initial point [61]. An animal’s hair, fleece and hide can be contaminated [62,63]. The lack of protection skills in veterinary medicine students also increases the risk when working with animals. So the direct or indirect exposure to contaminated aerosols is the main transmission route to humans [64]. But it is known that the ingestion of unpasteurized or raw milk from infected animals can be a mechanism of transmission [65,66]. Therefore, the consumption of raw milk or milk products could be a potential reason for the high seropositivity among our participants. But this is a hypothesis that should be analyzed in a future project. Transmission by tick bites is uncommon, but not impossible [67,68]. So a potentially higher tick exposure and bites among veterinarian students could partly explain the high seroprevalence.

The elevated prevalence of anti-*C. burnetii* IgM phase II (+) antibodies among students in the Trakia University suggests a direct correlation with their frequent contact with ruminants. The practical training involving hands-on livestock handling and farming activities contributes to the higher risk of exposure to *C. burnetii*. This cohort of students exhibited the highest seropositivity for anti-*C. burnetii* phase II IgG (+) antibodies. The cursus at Trakia University includes a number of practical classes involving farm animals (sheep, goats, cattle, horses, etc., including pregnant animals and animals that give birth), leading to an increased exposure to *C. burnetii* and a higher risk of infection. In contrast, the practical training at the University of Forestry is focused on domestic pets (mainly dogs and cats), which represents a lower epidemiological risk and may explain to some extent the lower seroprevalence in students from this university.

Our study revealed a higher seropositivity rate among male students as compared to females, with 32.8% of males testing positive for anti-*C. burnetii* phase II IgG antibodies versus 16.0% of females. The disparity by sex is in agreement with previous observations of ours [9] and suggests that male students might have had more intense exposure to risk factors during their practical training. For example, in the two Bulgarian veterinary medicine faculties, males more often participate in the birth of large animals such as cows, sheep and goats, whereas females commonly work with smaller animals such as dogs and cats. Consequently, males have an increased risk of direct exposure to infected materials. The detection of *C. burnetii* DNA in some blood samples implies that some students were experiencing early-phase infections. These findings are critical for early diagnosis and prompt treatment to prevent the progression towards chronic Q fever. The laboratory diagnosis of acute Q fever is based on detection via a PCR assay and serology methods [69]. Positive PCR results have identified patients with suspected early acute Q fever, since DNA is detectable within the 2 weeks after the start of symptoms, before or just as an anti-*C. burnetii* phase II IgM antibody response has been mounted [70]. Since the *C. burnetii* DNA detection time is always short for the diagnosis of acute Q fever, especially in regions of endemicity, this must be confirmed with a significant rise in titers specific to *C. burnetii* phase II antibodies in paired serum samples [71].

This study emphasizes the need for improved safety protocols and infection control measures in veterinary medicine training programs. Educating students about the risks and implementing strategies to reduce exposure to *C. burnetii* are essential steps in safeguarding their health. In this regard, the following protection measures against *C. burnetii* infection in veterinary medicine students can be mentioned in several respects [72,73,74]:

- Experimental animals in veterinary laboratories/farms must be placed in separate rooms;
- Pregnant infected animals (sheep, goats, cows, etc.) should not be used for practical student work;
- Mandatory biosafety procedures must be strictly followed in the microbiological and biomedical laboratories of Faculties of Veterinary Medicine;
- Placentas, aborted fetuses, other birth products and waste must be buried deeply using appropriate personal protective equipment (PPE); also, do not permit the eating of placenta by animals;
- Infectious waste must be sterilized and disposed at a specialized facility;
- All working surfaces must be disinfected against bodily fluids and animal dust;
- Wearing respiratory protective equipment and gloves is essential while handling animals when giving birth or in case of miscarriage.

This study has some limitations that need to be mentioned. First, the research involved a small number of participants. Second, we present results from the serological and PCR assays but we did not have access to data on the associated risk factors. Third, the participants under study did not present with clinical signs and symptoms of Q fever; i.e., all students were clinically healthy, so laboratory tests and imaging investigations were not performed. Despite these limitations, this research has several merits. To the best of our knowledge, this is the first study to presents serological evidence of *C. burnetii* infection among veterinary medicine students in Bulgaria. In addition, we performed a PCR analysis of the serum samples studied. These are important contributions to the overall knowledge of this infection in Bulgaria and the region of Southeastern Europe (the Balkan Peninsula).

## 5. Conclusions

In this study, we present data on the prevalence of *C. burnetii* infection among an at risk group (veterinary medicine students) in Bulgaria. We found high seroprevalence rates of this pathogen (30.8%) in the participants under study. This demonstrates that veterinarians in our country are exposed to an increased risk of Q fever, which can occur as an acute disease or a chronic infection. These findings require national health authorities to take adequate measures for the control and surveillance of this disease among groups at risk. In this direction, an important step is to increase the awareness of the veterinary community in Bulgaria regarding the measures that can be taken for Q fever prevention.

## Figures and Tables

**Table 1:** Distribution of veterinary students exposed and unexposed to *C. burnetii*, assessed by immune status or pathogen DNA identification by PCR in the venous blood, categorized by sex and location.

City	Sex	IgM (−)	IgM (−)	IgM (+)	IgM (+)	IgM (+)	IgM (+)
		IgG (−)	IgG (+)	IgG (−)	IgG (−)	IgG (+)	IgG (+)
		PCR (−)	PCR (−)	PCR (−)	PCR (+)	PCR (−)	PCR (+)
Sofia	Males	16	5	-	-	-	-
	Females	31	1	2	1	2	3
Stara Zagora	Males	19	15	-	1	2	1
	Females	64	7	8	4	2	1

Note: “+”, positive result; “−”, negative result.

**Table 2:** Association between *C. burnetii* exposure with sex and location variables. Relative risk for each experimental group is calculated.

Variables	Total, n	Exposed, n (%)	Unexposed, n (%)	Chi Square Statistics (df)	*p*-Value	Relative Risk (%)
**Pooled Sex**						
Males	59	24 (40.6)	35 (59.4)	2.22 (1)	<0.05	41
Females	126	31 (24.6)	95 (75.4)			25
**Pooled Location**						
Sofia	61	14 (23.0)	47 (77.0)	4.00 (1)	<0.05	23
Stara Zagora	124	41 (33.1)	83 (66.9)			33
**Males**						
Sofia	21	5 (23.8)	16 (76.2)	3.89 (1)	<0.05	24
Stara Zagora	38	19 (50.0)	19 (50.0)			50
**Females**						
Sofia	40	9 (22.5)	31 (77.5)	0.14 (1)	>0.1	23
Stara Zagora	86	22 (25.6)	64 (74.4)			26

## Data Availability

Dataset available on request from the authors.
